# Severe Catatonia in Early-Onset Schizophrenia Successfully Treated With Lorazepam and Aripiprazole in a 16-Year-Old Girl: A Case Report

**DOI:** 10.7759/cureus.89413

**Published:** 2025-08-05

**Authors:** Ghizlane Khaloui, Salma Abchir, Amal Mabkhout, Linda Rachidi, Ghizlane Benjelloun

**Affiliations:** 1 Child and Adolescent Psychiatry Department, Abderrahim Harouchi Mother-Child Hospital, Ibn Rochd University Hospital Center, Laboratory of Clinical Neurosciences and Mental Health, Faculty of Medicine and Pharmacy, Hassan II University, Casablanca, MAR

**Keywords:** adolescent, aripiprazole, benzodiazepines, catatonia, early-onset schizophrenia, neuroleptic malignant syndrome

## Abstract

Catatonia is a severe but often underrecognized neuropsychiatric condition in adolescents. We present the case of a 16-year-old girl with early-onset schizophrenia who developed acute catatonic symptoms during a relapse following risperidone reintroduction. The clinical presentation raised a diagnostic dilemma with neuroleptic malignant syndrome (NMS). A lorazepam trial led to partial improvement, but symptom resolution occurred only after adding low-dose aripiprazole. This case underscores the importance of early identification of catatonia in adolescents, careful differentiation from NMS, and the potential benefit of combined lorazepam and aripiprazole treatment in catatonic schizophrenia.

## Introduction

Catatonia is a severe neuropsychiatric syndrome characterized by a range of motor, behavioral, and affective disturbances. Though traditionally associated with schizophrenia, it is now recognized across a spectrum of psychiatric and medical conditions, including mood disorders, autism spectrum disorder, and autoimmune encephalitis [1]. This shift in understanding has significantly broadened the clinical relevance of catatonia across disciplines.

In pediatric populations, catatonia remains underrecognized and underdiagnosed. Several factors contribute to this, including the overlap of catatonic features with symptoms of other neurodevelopmental or psychiatric disorders (such as mutism, stereotypies, or withdrawal), limited awareness among clinicians, and hesitancy to apply adult-based diagnostic frameworks in younger patients [2]. This diagnostic gap poses a serious concern, as catatonia in children and adolescents can be equally severe and disabling as in adults.

Early identification of catatonia is critical, as untreated cases can progress to life-threatening malignant catatonia, characterized by autonomic instability, hyperthermia, and potential multiorgan failure [3]. The Bush-Francis Catatonia Rating Scale (BFCRS) remains a key tool for diagnosis, yet its routine use in children and adolescents remains inconsistent [4].

First-line treatment for catatonia involves high-dose benzodiazepines, particularly lorazepam, which often produce rapid and dramatic improvement in symptoms [5]. However, in cases where benzodiazepine response is incomplete, or electroconvulsive therapy (ECT) is contraindicated or unavailable, alternative pharmacological strategies must be considered. Second-generation antipsychotics such as aripiprazole, with partial dopamine agonist properties, have shown promise in treating catatonia associated with psychotic disorders, although data remain limited and mostly case-based [6,7].

This case report highlights a diagnostically challenging presentation of catatonia in an adolescent with early-onset schizophrenia. It illustrates the clinical course, including the therapeutic response to lorazepam followed by adjunctive aripiprazole, and underscores the importance of rapid intervention in optimizing both functional and psychiatric outcomes.

## Case presentation

A 16-year-old Moroccan female adolescent with a prior diagnosis of early-onset schizophrenia at the age of 14 was followed irregularly in our child and adolescent psychiatry department. Her family's psychiatric history was notable for a maternal aunt treated for postpartum depression and a paternal uncle with a psychotic disorder. Her perinatal and developmental history was unremarkable.

Her psychiatric symptoms began at age 14, initially characterized by progressive social withdrawal, clinophilia, affective lability, cognitive decline, academic difficulties, and marked functional impairment. After approximately five months, she developed persecutory delusions targeting family members and classmates, auditory, visual, and cenesthetic hallucinations, conceptual and behavioral disorganization, along with marked anxiety and sleep-onset insomnia. Symptom severity gradually increased, leading to significant functional impairment.

An initial emergency pediatric assessment excluded any underlying medical or toxic causes. She was referred to our department, where an intensive outpatient follow-up was initiated due to the unavailability of inpatient beds. Early-onset schizophrenia was diagnosed according to the Diagnostic and Statistical Manual of Mental Disorders, Fifth Edition (DSM-5) [8]. A pretreatment evaluation, including laboratory tests (complete blood count (CBC), serum electrolytes, liver and renal function tests) and an electrocardiogram (ECG), was normal. Risperidone was initiated at a low dose and gradually titrated up to 1.5 mg/day, resulting in good tolerability and improvement of psychotic symptoms. However, she was lost to follow-up after three months of treatment.

Five months later, she was urgently referred due to a relapse with the resurgence of hallucinations, persecutory delusions, and severe anxiety. Clinical examination revealed a highly anxious and distressed adolescent, with limited speech, at times disorganized, along with auditory and visual hallucinations. A psychotic relapse was diagnosed, triggered by discontinuation of medication and precipitated by academic stress without educational accommodations.

The patient was admitted for full-time inpatient care in the adolescent unit of the child and adolescent psychiatry department at Ibn Rochd University Hospital in Casablanca. Admission work-up, including laboratory tests, toxicology screening, and ECG, was unremarkable.

Given her previous positive response to risperidone, the medication was reinitiated at 0.5 mg once daily on day one and titrated to 1 mg twice daily on day two. On day three, she developed generalized rigidity without fever or altered levels of consciousness. Neuroleptic malignant syndrome (NMS) was initially suspected; risperidone was promptly discontinued, and serum creatine kinase (CK) levels were obtained, which were within normal limits. Within hours, she exhibited marked psychomotor retardation, stupor, negativism, prolonged posturing with catalepsy, and refusal to swallow saliva, resulting in hypersalivation. She was barely able to write down persecutory delusions and reported auditory hallucinations while crying. She rapidly became mute, maintained a fixed gaze with a persistent anguished facial expression, was unresponsive to verbal reassurance, and refused to eat.

A diagnosis of catatonia was considered according to DSM-5 criteria, and the Bush-Francis Catatonia Rating Scale (BFCRS) was administered [9], yielding a score of 32. Risperidone was withheld, and oral lorazepam (1 mg) was administered on day four as both a diagnostic and therapeutic trial. Within hours, a marked reduction in rigidity was observed, supporting the diagnosis of catatonia and indicating a positive response to lorazepam.

In light of this favorable response, the lorazepam dosage was rapidly increased to 6.5 mg three times daily under close monitoring. Additional investigations, including brain magnetic resonance imaging (MRI) and electroencephalogram (EEG), were within normal limits. Testing for anti-N-methyl-D-aspartate receptor antibodies was requested but could not be performed due to financial constraints.

Over the following 15 days, catatonic symptoms gradually improved, with the BFCRS score decreasing to 17. However, further progress slowed, with stagnation at 15. Prominent symptoms included mutism, negativism, posturing, and social withdrawal. In parallel, psychotic features became more pronounced, including a persecutory delusion, auditory and visual hallucinations, disorganization, and bizarre behavior. ECT was considered but declined by the parents due to concerns about potential memory side effects.

On day 40 of hospitalization, low-dose aripiprazole (2.5 mg once daily) was introduced, considering its partial agonist-antagonist dopaminergic profile. Improvement was observed after 48 hours and became more significant following a dose increase to 5 mg twice daily on day 13 of aripiprazole treatment (hospital day 53), with a reduction in mutism, negativism, and withdrawal. The BFCRS score decreased to 9. On day 19 of aripiprazole treatment (hospital day 59), she experienced fine resting tremors in the upper limbs, which resolved spontaneously after 72 hours. The dose was increased to 7.5 mg three times daily on day 25 (hospital day 65). A progressive and marked improvement was noted, with the BFCRS score reaching 2 by day 36 (hospital day 76), residual posturing and fixed gaze only. She became increasingly interactive, followed instructions, participated in therapeutic workshops, and exhibited more coherent speech. Notably, each dosage increase led to a clear clinical benefit within 24-48 hours, followed by a plateau until the next titration. On day 33 (hospital day 73), aripiprazole was increased to 10 mg three times daily, and by day 36, the catatonic syndrome had fully remitted. At 12.5 mg three times daily (day 39 of aripiprazole treatment, hospital day 79), psychotic symptoms also showed near-complete remission, with only mild residual cognitive deficits.

Gradual tapering of lorazepam was initiated on day 59 of hospitalization, with weekly dose reductions of 0.5 mg, then every three to four days. It was completely discontinued after one month and 20 days, with no recurrence of catatonia.

After four months of full-time inpatient care, the patient was transitioned to partial hospitalization with ongoing clinical improvement. A gradual return to school was facilitated with appropriate educational accommodations, and full reintegration was achieved after one month.

She was discharged after five months of hospitalization, maintained on aripiprazole 12.5 mg three times daily, with close outpatient psychiatric follow-up and referral for cognitive remediation therapy. At the six-month follow-up, the patient maintained good clinical status, with further improvement in cognitive symptoms.

## Discussion

This research highlights several key clinical considerations regarding catatonia in pediatric populations, particularly in adolescents with schizophrenia: (1) the importance of differentiating catatonia from NMS in patients presenting with psychomotor symptoms following antipsychotic use, (2) the potential for partial or incomplete response to benzodiazepines alone, and (3) the clinical utility of aripiprazole as a safe and effective adjunctive treatment in catatonia associated with early-onset schizophrenia.

Differential diagnosis

Catatonia remains a diagnostically and therapeutically challenging neuropsychiatric syndrome, particularly in children and adolescents, where it is often underrecognized or misdiagnosed as primary psychosis, mood disorders, or neurodevelopmental conditions [2,10]. Its clinical features may overlap with the negative symptoms of schizophrenia, severe depressive states, or adverse effects of medications, complicating timely diagnosis [11]. In the present case, catatonic symptoms emerged following the reintroduction of risperidone, initially raising concerns for NMS, a potentially life-threatening condition. However, the absence of hyperthermia, normal CK levels, preserved consciousness, and the rapid response to lorazepam supported a diagnosis of catatonia over NMS [12].

Treatment challenges and responses

Benzodiazepines, particularly lorazepam, remain the first-line treatment for catatonia, often resulting in significant clinical improvement. Nevertheless, an incomplete response is observed in approximately 20-40% of cases [13]. ECT, although highly effective and considered the gold standard for refractory catatonia, remains underutilized in pediatric populations due to ethical concerns, limited availability, and apprehension regarding potential cognitive side effects [14].

In our case, despite an initial response to lorazepam, symptom improvement plateaued, necessitating consideration of adjunctive strategies. In this context, aripiprazole was introduced and led to a sustained resolution of both catatonic and psychotic features. Traditionally, antipsychotics have been avoided in acute catatonia due to the risk of exacerbating motor symptoms or triggering NMS. However, emerging evidence supports the cautious use of certain second-generation antipsychotics, particularly in catatonia associated with psychotic disorders. A 2025 systematic review involving 110 patients with catatonic schizophrenia or mood disorders reported clinical benefits with antipsychotic use in approximately 60% of cases, especially with agents such as aripiprazole and olanzapine, which demonstrated more favorable safety profiles than first-generation antipsychotics [7,15].

Aripiprazole’s distinctive pharmacological properties, partial agonism at dopamine D₂ and serotonin 5-HT₁A receptors, and antagonism at 5-HT₂A receptors, may help rebalance dopaminergic and serotonergic pathways, potentially alleviating catatonic symptoms without exacerbating motor abnormalities [16,17]. Recent case reports have also shown its effectiveness [18-20]. Additionally, olanzapine has demonstrated efficacy in pediatric catatonic schizophrenia in small observational studies, offering an alternative therapeutic option in selected cases [21,22].

Implications for practice

Beyond pharmacological management, this case highlights the ongoing challenges related to the timely recognition and comprehensive care of pediatric catatonia. Limited awareness among clinicians, the absence of standardized pediatric protocols, and restricted access to certain treatments, such as ECT, contribute to potential delays and suboptimal outcomes. Strengthening interdisciplinary collaboration and integrating routine screening for catatonic features into psychiatric assessments could facilitate earlier diagnosis and tailored interventions, ultimately improving prognosis in this vulnerable population.

## Conclusions

This research highlights the diagnostic and therapeutic challenges of catatonia in adolescents with schizophrenia, particularly the difficulty in differentiating it from NMS and the potential for incomplete response to benzodiazepines. Prompt recognition and initiation of appropriate treatment are essential to prevent severe complications and improve outcomes.

The clinically significant remission observed with adjunctive aripiprazole in this patient suggests that second-generation antipsychotics, particularly those with partial dopamine agonist properties, may play a valuable role in managing refractory catatonia when standard therapies are insufficient. Further research is warranted to clarify their place in treatment algorithms for pediatric catatonia.
